# The cerebral and cognitive changes after intermittent theta burst stimulation (iTBS) treatment for depression: study protocol for a randomized double-blind sham-controlled trial

**DOI:** 10.1186/s13063-024-08606-8

**Published:** 2024-11-11

**Authors:** Marte C. Ørbo, Sabine Høier, Torgil R. Vangberg, Gabor Csifcsak, Ole K. Grønli, Per M. Aslaksen

**Affiliations:** 1 Department of Psychology, Faculty of Health Sciences, UiT – the Arctic University of Norway, Tromsø, Norway; 2 Department of Clinical Medicine, Faculty of Health Sciences, UiT – the Arctic University of Norway, Tromsø, Norway; 3 The PET Imaging Center, University Hospital of North Norway, Tromsø, Norway; 4 Department of Psychology, UiT – the Arctic University of Norway, Tromsø, Norway; 5 Division of Mental Health and Substance Abuse, University Hospital of North Norway, Tromsø, Norway; 6 Regional Centre for Eating Disorders, University Hospital of North Norway, Tromsø, Norway

**Keywords:** Depression, Neuroimaging, Theta burst, Cognition, fMRI, White matter, FreeSurfer

## Abstract

**Background:**

The therapeutic use of intermittent theta burst stimulation (iTBS) delivered to the left dorsolateral prefrontal cortex (LDLPFC) is a relatively new but promising treatment option for depression. There is a need for more knowledge on the mechanisms involved in its antidepressant effects.

**Methods:**

This is a single-centre, prospective, randomized, double-blind, placebo-controlled trial with two arms, iTBS and sham iTBS. Adult outpatients with unipolar major depressive disorder of at least moderate severity will undergo cognitive assessment with an N-back task (0-back and 2-back), functional and structural magnetic resonance imaging and assessment of depression severity before and after brain stimulation. Neuronavigated iTBS or sham stimulation will be targeted at the LDPFC once a day for 10 consecutive workdays. ITBS will be delivered with the parameters 120% of resting motor threshold, triplet 50 Hz bursts repeated at 5 Hz; 2 s on and 8 s off, 600 pulses per session with a total duration of 3 min 9 s. The severity of depression will be measured with the Montogomery Aasberg Depression Rating Scale and the Beck Depression Inventory – second edition.

In the iTBS group relative to sham, we expect significant antidepressant effects and improved N-back performance, associated with increased integrity in white matter tracts functionally connected with the LDLPFC and emotion regulation areas within the rostral anterior cingulate cortices, alongside potential increases in cortical thickness in these regions. On functional imaging, we expect to observe increased brain activity in the LDPFC during the performance of the N-back condition with higher cognitive load (2-back) in the iTBS group relative to sham.

**Discussion:**

iTBS is a promising, time-efficient, and considered a safe treatment option for depression according to existing evidence. This trial aims to assess the neurocognitive impact of a 2-week, once-daily iTBS compared to sham iTBS, targeting the LDLPFC in depressed adult outpatients. The study investigates the relationships between changes in cerebral measures and cognitive performance on an N-back task in relation to the antidepressant effect following iTBS. This trial delves into the neurocognitive mechanisms of iTBS in depression, potentially offering novel scientific insights into its treatment effects and mechanisms of action.

**Trial registration:**

ClinicalTrials.gov NCT06534684. Retrospectively registered on August 1st 2024.

## Administrative information

Note: the numbers in curly brackets in this protocol refer to SPIRIT checklist item numbers. The order of the items has been modified to group similar items (see http://www.equator-network.org/reporting-guidelines/spirit-2013-statement-defining-standard-protocol-items-for-clinical-trials/).

**Table:** 

Title {1}	The cerebral and cognitive changes after intermittent theta burst stimulation (iTBS) treatment for depression: study protocol for a randomized double-blind sham-controlled trial.
Trial registration {2a and 2b}.	Retrospectively registered in Clinical Trials NCT06534684. August 1st 2024. https://clinicaltrials.gov/study/NCT06534684
Protocol version {3}	First submitted to ClinicalTrials.gov: 2024–05-25, first posted:2024–08-02, last update posted 2024–08-02, Last verified:2024–08
Funding {4}	PhD Grant from the Northern Norway Regional Health Authority, grant number HNF1671-23.
Author details {5a}	Marte C. Ørbo. Department of Psychology, Faculty of Health Sciences, UiT – the Arctic University of Norway, Tromsø, Norway Sabine Høier. Department of Psychology, Faculty of Health Sciences, UiT – the Arctic University of Norway, Tromsø, Norway Torgil R. Vangberg. Department of Clinical Medicine, Faculty of Health Sciences, UiT – the Arctic University of Norway, Tromsø, Norway. The PET Imaging Center, University Hospital of North Norway, Tromsø, Norway. Gabor Csifcsak. Department of Psychology, UiT – the Arctic University of Norway, Tromsø, Norway Ole K. Grønli. Department of Clinical Medicine, Faculty of Health Sciences, UiT – the Arctic University of Norway, Tromsø, Norway. Division of Mental Health and Substance Abuse, University Hospital of North Norway, Tromsø, Norway Per M. Aslaksen. Department of Psyhology, Faculty of Health Sciences, UiT – the Arctic University of Norway, Tromsø, Norway. Regional Centre for Eating Disorders, University Hospital of North Norway, Tromsø, Norway.
Name and contact information for the trial sponsor {5b	Northern Norway Regional Health Authority, Email: forskningsmidler@unn.no
Role of sponsor {5c}	The sponsor has no role in study design, collection, management, analysis and interpretation of data, written the report, submit the report for publication, and has no authority over any of these activities

## Introduction

### Background and rationale {6a}

Depressive disorders impose substantial societal and economic burdens, with conventional treatments often inadequate for many patients, underscoring the necessity to explore alternative therapeutic strategies [1, 2]. Non-invasive brain stimulation techniques, such as intermittent theta burst stimulation (iTBS) have emerged as promising interventions for depressive disorders, with the potential for cognitive improvement in addition to antidepressant effects [3–5]. This is important because cognitive dysfunction is involved in impaired functioning in depression and is associated with lack of remission and relapse [6].

iTBS, a modified form of high-frequency repetitive transcranial magnetic stimulation (HF rTMS), delivers targeted magnetic field pulses, inducing enduring changes in brain activity. Unlike the HF-rTMS sessions lasting approximately 20–40 min, iTBS protocols for depression are significantly shorter, lasting only about 3 min, thus offering reduced stimulation duration and enhanced patient comfort while maintaining therapeutic efficacy. The treatment is reported to be well-tolerated with only mild side effects [7–10].

In therapeutic use for depression, excitatory paradigms with rTMS or iTBS are most often targeted at the left dorsolateral prefrontal cortex (LDPFC) [11]. It is believed that the antidepressant mechanism of action involves a normalization of imbalanced activation patterns in the prefrontal cortex of depressed individuals [12]. iTBS operates through rapid bursts of pulses delivered at a frequency of 50 Hz, mimicking the brain’s theta rhythm [13]. This rhythm is associated with long-term potentiation and enduring brain changes primarily in the medial part of the prefrontal cortex (including the anterior cingulate cortex), regarded as important brain correlates of cognitive control processes [5]. Hence, iTBS has been hypothesized to improve cognitive control functions in depression alongside amelioration of mood symptoms [14].

However, much remains unknown about the antidepressant mechanisms of action of iTBS and the neurocognitive changes induced by iTBS [15]. Hence, this trial aims to assess the effect of iTBS targeted to the LDLPFC on improvements in frontal lobe function in depression. Previous studies on cerebral anatomical changes after TMS in depression have not used the iTBS protocol. Nevertheless, using 10 Hz rTMS Boes et al. [16] found that the rostral cingulate cortex increased in thickness following traditional rTMS treatment and that the cerebral thickness of this area before starting treatment was a marker for treatment response. Regarding white matter structural changes, prior studies have implicated decreased fractional anisotropy (FA) in white matter tracts, more specifically in the uncinate fasciculus and in the cingulum bundle, as a characteristic of major depression. Using traditional rTMS, FA has shown to increase in depressed patients. First, FA increase was shown in the left middle frontal gyrus and the change correlated with the decrease in depressive symptoms [17]. Furthermore, Tateishi et al. [18] found increased white matter integrity after rTMS in depressed patients, as well as improvements on neurocognitive tests. However, neurocognitive improvement was not correlated to FA increase [18]. To our knowledge, there are no studies examining similar structural changes in depressed brains following iTBS. Moreover, it is conceivable that iTBS may improve neurocognitive functions, given the crucial role of the frontal cortices for cognition in general, and executive functions (EF) specifically. The N-back task is frequently utilized to assess working memory (WM) and EF deficits [19]. Given the anticipated heightened neural activity in the left DLPFC induced by iTBS, employing the N-back task during brain functional magnetic resonance scanning (fMRI) could serve as a reliable indicator of this neural modulation, given its established association with DLPFC activity [19].

### Objectives {7}

The primary objectives of the present trial are to assess the impact of a 2-week, once-a-day application of iTBS over the LDLPFC on cerebral and N-back task performance, and to examine the associations between changes in cerebral functions, cognition, and the reduction in depression symptoms following iTBS. The underlying hypothesis posits that the reduction in scores on the Montgomery-Åsberg Depression Rating Scale (MADRS) [20] and Beck Depression Inventory-II (BDI-II) [21], will be statistically significant after the 2-week iTBS treatment in comparison to sham treatment, drawing from data reported in prior studies [8]. Although a decrease in depressive symptoms is expected in the sham group due to the placebo effects [22] we anticipate that the physiological effects of iTBS will surpass placebo responses within the parameters of the current study design [23].

Research questions and hypotheses:

1. Does the iTBS protocol induce significant changes in the integrity of white matter cerebral tracts and are these changes related to reductions in depression and improved N-back task performance? We expect that the reduction in MADRS and BDI-II scores from baseline to the post-test will be significantly associated with changes in white matter fractional anisotropy (FA) induced by iTBS, where a larger increase in FA will show larger reductions in depressive symptoms in the iTBS group compared to the group receiving sham.
2. Are changes in depressive symptoms and executive functions after iTBS treatment related to grey matter anatomical changes in cerebral measures? The expected larger reductions in MADRS and improved N-back task performance in the iTBS group between baseline and post-test compared to the placebo group will be significantly associated with increased thickness of the rostral anterior cingulate cortex and the LDPFC.
3. Will iTBS treatment change the observed neural efficacy and behavioural performance to cognitive demanding tasks (N-back) in depressed patients? We expect that patients who received iTBS treatment will display significantly larger changes in functional BOLD response, with significantly higher BOLD response in the DPFC to a demanding cognitive task (2-back) during fMRI scanning compared to patients receiving sham stimulation. Larger reductions in depressive symptoms will be associated with larger changes in fMRI BOLD responses and greater improvements on the N-back task.

### Trial design {8}

This trial is a single-centre, randomized, sham-controlled, parallel-group, double-blind clinical trial. Participants will be recruited prospectively.

## Methods: participants, interventions, and outcomes

### Study setting {9}

The trial will be performed in clinical laboratories at the UIT, the Artic University of Norway, and the University Hospital of North Norway—Health Trust (UNN-HF), Tromsø, Norway. The laboratories at UIT and the UNN are located in the same campus area.

### Eligibility criteria {10}

Inclusion criteria are adult out-patients between 22–65 years of age meeting the diagnostic criteria of at least moderate depression. Fluency in the Norwegian language is required. Alongside the M.I.N.I. psychiatric interview [24], the MADRS [20] will be administrated. A MADRS score of ≥ 20, usually indicates a moderate depression. Patients must volunteer to provide informed, written consent, be able to follow the treatment schedule and have a satisfactory safety screening for iTBS and MRI. Furthermore, drug therapy must have been stable for the last 3 weeks before the first treatment day with iTBS and is to be kept stable throughout the study until close-out, 4 weeks after the last day of iTBS treatment.

Patients will be excluded from the study if the clinical interview indicates that the current depressive episode is in the mild range or triggered by grief or a recent major stressful life event, or contrary, that the current episode fulfils the criteria for a major depressive episode requiring inpatient treatment and heightened risk of suicide. Further exclusion criteria are: The clinical assessment indicates the likelihood of bipolar disorder, borderline personality disorder or psychotic symptoms, alcohol or substance abuse/addiction in the last 6 months, current eating disorders, obsessive–compulsive disorder, or post-traumatic stress disorder. Exclusion criteria due to safety issues are a medical history of seizure, neurological or neurosurgical pathologies, cardiac or systemic disease, metallic prosthetic material or foreign objects (pacemakers, prosthetic eye equipment, etc.), and being pregnant or currently breast-feeding [25, 26]. Eligibility interviews are conducted by a medical doctor or a specialist in clinical psychology.

### Who will take informed consent? {26a}

Prior to enrolment, detailed information about the study is provided, an eligibility screening (safety check, inclusion/exclusion criteria) is conducted by telephone, and the consent form is sent by e-mail. This procedure is conducted by one of the main investigators. A medical doctor is consulted in case of patient’s medications, and possible interactions with iTBS treatment. If an eligible patient still wishes to participate after this step, the written informed consent form must be handed in at the study site prior to the baseline assessments.

### Additional consent provisions for collection and use of participant data and biological specimens {26b}

All participants will donate blood samples for a biobank in a related, ongoing study with its own ethical approval and associated published protocol [23], but none of the samples will be used for studies described in the present protocol.

## Interventions

### Explanation for the choice of comparators {6b}

In this RCT, the effects of iTBS will be compared against sham iTBS. Sham iTBS resembles the active iTBS condition and provides a sensory experience that is difficult to separate from real iTBS for naïve participants. The use of a placebo treatment will allow us to separate the effect of iTBS from other non-iTBS-specific factors.

### Intervention description {11a}

Theta burst stimulation will be performed with a Mag & More PowerMag EEG 100 system (https://magandmore.com) with a double PMD70 p-cool figure-of-eight coil. Sham stimulation will be performed by the double PMD70 p-cool figure-of-eight coil Sham system. The sham system has an identical look, weight and sound compared to the active coil, and delivers electrical stimulation that can be felt at the skin but without penetrating the skull. Resting-motor threshold (RMT) will be determined prior to stimulation in accordance with the consensus guidelines for rTMS [21]. ITBS will be delivered with the same parameters as in Blumberger et al., [8]: 120% RMT triplet 50 Hz bursts repeated at 5 Hz; 2 s on and 8 s off, 600 pulses per session with a total duration of 3 min 9 s. Treatment will be provided for 10 days for two consecutive weeks (except Saturdays and Sundays). Before the first treatment, participants will perform MRI examinations (T1, DTI, fMRI) and the high-resolution T1-weighted obtained at that point will be used for real-time MRI-guided neuro-navigation for optimal coil positioning. A Localite TMS-navigator system (https://www.localite.de) will be used for the determination of the target area within the DLPFC. The Localite system visualizes the patient’s brain on a pc-screen based on the T1 images, and the coil is connected to the Localite system making it possible to move the coil over the desired brain structure and correctly position it. The target MNI coordinate will be *x* − 38, *y* + 44, *z* + 26, based on the findings of Fox et al. [12] who identified this point based on clinical outcomes and whole-brain functional connectivity for rTMS studies.

### Criteria for discontinuing or modifying allocated interventions {11b}

The criteria for discontinuing or modifying allocated interventions for a given participant include the following: worsening of disease, report of adverse side effects, withdrawal of consent, change in drugs or not receiving stimulation on more than 2 days. If the patient is absent from one or two stimulations, these will be added to the treatment schedule. For safety reasons, participants will be asked about sleep, alcohol, illegal drugs and medication use prior to each simulation. Participants will be asked about their well-being prior to and after each iTBS stimulation. The participants will be asked to drink no more than one unit of alcohol the night before stimulation. If the patient reports drinking more alcohol, stimulation will be postponed to the day after. Stimulation will also be postponed if the participant reports significant tiredness and/or lack of sleep the night before [23].

### Strategies to improve adherence to interventions {11c}

Patients are carefully informed about the requirements for participation prior to study enrolment. During participation, they are cared for by the study nurses on a day-to-day basis. A clinical psychologist assesses the patient’s well-being after five stimulations.

### Relevant concomitant care permitted or prohibited during the trial {11d}

No treatments are prohibited during the trial, but drug therapy must have been stable for the last 3 weeks prior to the first treatment day with iTBS and must be kept stable throughout the study until 4 weeks after the last day of iTBS treatment. All concurrent treatments will be recorded [23].

### Provisions for post-trial care {30}

A total of ten sessions of once-a-day iTBS is offered to a patient who has received placebo and still meets the inclusion criteria at the time of disclosure of allocation.

### Outcomes {12}

The primary outcome measures of this trial are cerebral changes and their association with changes in depressive symptoms and cognitive performance on the N-back task after 2 weeks of daily iTBS or sham iTBS.

Participants will undergo brain imaging prior to and after the iTBS or sham protocol. T1-weighted anatomical cerebral MRI images will be obtained for measures of cortical volume and thickness. Diffusion tensor imaging (DTI) will be used to get data for white matter integrity, whereas fMRI data will be used to obtain functional blood-oxygen-level-dependent (BOLD) data for responses to the N-back task during scanning.

Change in level of depression in measured pre and post-stimulation with the semi-structured clinician-led patient interview MADRS [20] and with the patient-reported BDI-II [21]. Based on the data from Blumberger et al. [8], we estimate a mean decrease of approx. 25% in depressive symptoms over a 2-week treatment course in those receiving iTBS. The minimal clinically important difference (MCID) for BDI-II is suggested to be 17.5% change from baseline and 13% change for the MADRS [27].

Between-group change in working memory and executive functions is measured with performance on a N-back task prior to and after stimulation. The N-back task can be performed on a laptop prior to and after the iTBS stimulation protocol and it is also implemented for use in fMRI [28] We will use 0-back and 2-back tasks that generate measures of the number of correct responses, reaction time, commission and omission errors.

### Participant timeline {13}

**Fig. 1:**
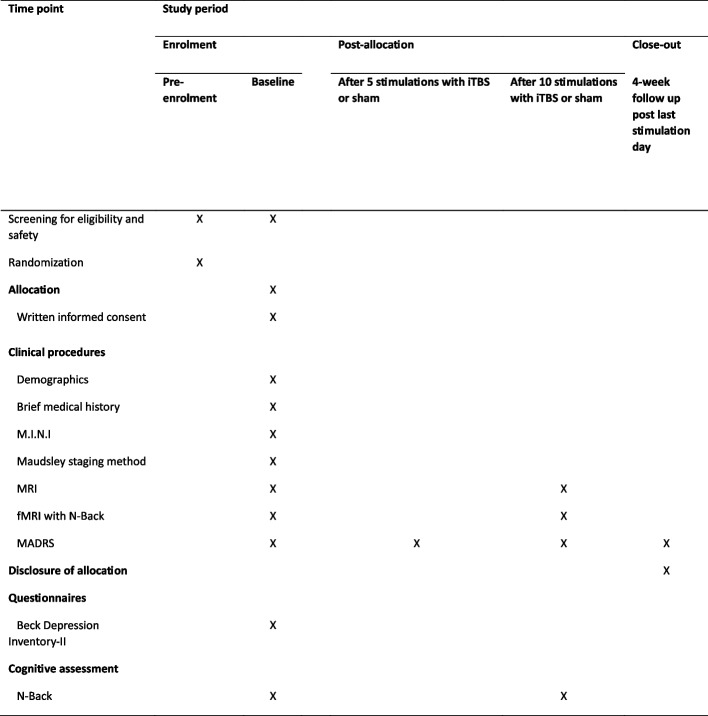
Schedule of enrolment, interventions and assessments. *A*bbreviations: *M.I.N.I* Mini International Neuropsychiatric Interview, *MADRS* Montgomery and Asberg Depression Rating Scale, *fMR*I functional magnetic resonance imaging

Figure 1 shows the schedule of enrolment, interventions and assessments.

### Sample size {14}

To date, only a single study has examined the relation between fractional anisotropy (FA) and iTBS [29], however, that study tested the after-effects of iTBS in the primary motor cortex and included only healthy participants. To our knowledge, there are no studies to date that have examined the change in FA after iTBS treatment. Furthermore, there are to our knowledge no previous studies that have measured the change in cortical thickness after iTBS treatment. Due to the lack of previous studies investigating changes in functional and structural cerebral measures after iTBS treatment in depressed patients, we based the sample size calculation on the expected cognitive improvement in N-back performance during fMRI. For the fMRI measures using the N-back task, we expect similar effect sizes as in previous comparable studies (summarized in [28]), where moderate to large effect sizes and significant group differences were observed with group sizes of 20–30 participants when using the same N-back stimuli as we do. Based on previous data [28] for N-back changes in fMRI, in a repeated analysis (*F*-test), medium effect size (*f* = 0.25), two groups, alpha = 0.05, and power = 0.90, correlation between repeated measures = 0.5, sphericity correction = 1, the required sample size is 46 for the fMRI analyses, with equivalent group sizes of iTBS and sham. There is limited information about drop-out rates for iTBS treatment, however, a meta-analysis on rTMS [30] suggested the mean drop-out rate to be between 7 and 8%. Thus, assuming a total drop-out of 10%, the present study must include a total of 50 participants.

### Recruitment {15}

The project group collaborates closely with the psychiatric outpatient clinic at UNN-HF, Tromsø. Written information about the project is sent to participants on the waiting list. The project has been communicated to all general practitioner offices in Tromsø as well as the community centres for low-threshold services for mental health support. Additionally, information about the project is distributed through multiple channels such as the local media on the radio and in newspapers and internal media at the hospital and university; the project has a website and physical posters have been hung around the city of Tromsø.

## Assignment of interventions: allocation

### Sequence generation {16a}

The RCT will be run with an allocation ratio of 1:1 to the two groups (iTBS and sham). We expect a 1:1 ratio of males to females since the groups will be stratified by gender in the randomization.

### Concealment mechanism {16b}

The randomization will be performed in MATLAB (https://se.mathworks.com/products/MATLAB.html), with the “randperm” command that performs randomization in four permuted blocks to groups and gender in a single operation. The a priori randomization procedure in MATLAB will be performed by a research assistant prior to the start of the study. This research assistant is not involved in the data collection.

The research assistant will produce numbered coloured envelopes (e.g. pink = female, blue = male), each containing a number indicating the type of coil to be used (e.g. 1 = active, 2 = sham).

### Implementation {16c}

The staff member who performs the stimulation procedure will open the envelope. The staff members who provide iTBS will not assess the patient. Clinicians who assess the patients will not be aware of the treatment group. Thus, neither the clinician who assesses the patient nor the patient will know which treatment is provided. A study identification code will be allocated to each participant upon entry. Their name or national identity number will not appear in the research data collected.

## Assignment of interventions: blinding

### Who will be blinded {17a}

The patient will be blinded. The assessor of the patients’ clinical symptoms will be blinded. The staff members who provide iTBS will not assess the patient. Clinicians who assess the patients will not be aware of the treatment group. Thus, neither the clinician who assesses the patient nor the patient will know which treatment is provided. Four weeks after the last stimulation day, treatment allocation will be disclosed to the patient.

### Procedure for unblinding if needed {17b}

Serious adverse events will make unblinding permissible. A serious event will also stop the data collection until the reason for the event is clarified.

## Data collection and management

### Plans for assessment and collection of outcomes {18a}

At baseline, prior to stimulation, a clinical psychologist or a medical doctor will perform a diagnostic assessment using the M.I.N.I [24] and the MADRS [20] in a clinical interview. Information obtained from the participant at baseline includes age, sex, marital status, length of education, living situation, history of depression and comorbidity. The Maudsley Staging Method [31] is used to score the level of treatment resistance. A visual N-back task [32] is performed at a laptop and the BDI-II [21] is a self-report pen-and-paper measure.

The Maudsley Staging Method is a multidimensional model developed to define and stage treatment resistance in unipolar depression. The model maps treatment failures, severity of symptoms and duration of depressive episodes into a continuous score with a cut-off for treatment resistance [31].

The severity of depression symptoms will be evaluated using MADRS and the BDI-II. The MADRS will be administrated pre- and post-stimulation as well as once midway through the stimulation protocol and at close out. BDI-II will be administrated pre- and post-stimulation and again prior to close-out. At all-time points, the outcomes are collected by experienced clinical psychologists or a psychiatrist.

MADRS is a widely used clinician-administered instrument for measuring the severity of depressive episodes in patients with mood disorders. It consists of 10 items rated on a scale of 0 to 6, where higher scores indicate a higher degree of symptoms. MADRS has shown good concurrent validity [33, 34]. The MADRS has shown high correlations with other well-known and widely used clinician-rated scales such as the Hamilton Depression Rating Scale [35], underscoring the MADRS high concurrent validity. Intra-rater reliability was originally reported to be excellent [20]. However, subsequent studies have reported varying intraclass correlation coefficients (ICC). Maier et al. showed total scale score ICCs of 0.73, 0.66 and 0.82 in three separate samples [36]. It has later been demonstrated that the use of experienced clinicians as raters and the use of a structured guide for rating increases the intra-rater reliability. Williams and Kobak found an intraclass correlation for the total scale score between raters on the MADRS of 0.93 [37] whereas Geijer et al. [38] found an inter-rater reliability of ICC = 0.95.

The Beck Depression Inventory-II (BDI-II) [21] is one of the most widely used self-report instruments for assessing the severity of depression. It consists of 21 items and measures the degree of depression in ages 13 years and above. The concurrent validity of the BDI-II with other instruments and scales used to assess the severity of depression symptoms ranges from adequate to high with correlations ranging from 0.66 to 0.86 [39].

An n-back task [32] will be used to assess aspects of working memory, executive functions, reaction time and sustained attention to visual stimuli. The N-back is a widely used cognitive task that requires participants to concurrently encode, maintain, and update stimuli presented *n* trials previously [19]. The present study uses a visual in-house version of the task created in the software Psychopy [40] and presented on a laptop during the lab testing and on the MRI-scanner computer screen. In the present study, we will use 0-back and 2-back tasks. The instruction for the 0-back is to push the spacebar (for laptop administration) or the response button when in the fMRI lab as fast as possible when the letter “X” is presented on the screen. All other letters should be ignored. For the 2-back task, the participants should push the spacebar if the letter presented on the screen matched the letter presented two letters prior. Letters from A to Z are randomly presented with 1000 ms stimulus and 2000 ms interstimulus intervals in both the 0-back and the 2-back task. Participants are allowed to respond during the stimulus presentation and for 1000 ms after the stimuli ended. A total of 10 trials (five 0-back, and five 2-back) are performed in each run with a fixed order of the 0-back preceding the 2-back task. Participants will be tested 2 times (pre-test and post-test) outside the MRI scanner and two times (pre- and post-test) in the scanner. Hence, the order of the n-back runs are (1) pre-test in lab, (2) pre-test in MRI scanner, (3) post-test in lab, and (4) posttest in MRI-scanner.

### Plans to promote participant retention and complete follow-up {18b}

If a patient withdraws from the treatment, the information collected before the withdrawal will be kept and used. If a patient withdraws his or her study consent, the information collected will not be used.

### Data management {19}

The data management plan for this project was reviewed and approved by the Regional Committee for Medical Research Ethics Northern Norway (REC), UiT The Arctic University of Norway, and the data protection officer at UNN-HF. All data will be stored on secure network locations governed by the University Hospital.

### Confidentiality {27}

A study identification code will be allocated to each participant upon entry. Their name, national identity number, or other information that can identify the participant directly will not appear in the research data collected, and the list of identification codes that can be used to identify participants who wish to view, delete or correct their own information or used in other cases where unblinding is needed, will be stored separately and only the project manager will have access to it. According to Norwegian legislation, the list of identification codes must be saved for 5 years after completion of the trial and the data will then be anonymized.

### Plans for collection, laboratory evaluation and storage of biological specimens for genetic or molecular analysis in this trial/future use {33}

Blood is drawn from the participants in the present study as described in a previous study protocol [23], but these data will not be used in the present trial.

## Statistical methods

### Statistical methods for primary and secondary outcomes {20a}

Repeated measures data will be analysed by linear mixed models (LMM) which allow for a combination of fixed and random effects. LMM estimates the parameters using all available patient data and is thus less sensitive to scattered missing data, and has flexible modelling options for the covariance structure of the data [41]. Single means (e.g. baseline data) will be analysed with statistical methods within the framework of general linear models (GLM), if the statistical assumptions for GLMs are met. In case of non-normal data, non-parametric statistics will be performed. SPSS and R will be used for statistical analyses of clinical and cognitive data. Analyses of brain imaging data will be performed in separate programs according to the type of data. FreeSurfer (FS) (https://surfer.nmr.mgh.harvard.edu/) will be used for preprocessing of structural imaging data (volumetric analyses and segmentation), and Tracula inbuilt in FS will be used for DTI data [42], and Permutation Analysis of Linear Models (PALM) will be used to perform statistics on structural data preprocessed in FS, and in analyses where imaging data is combined with other data as covariates or factors [43]. SPM12 (https://www.fil.ion.ucl.ac.uk/spm/software/spm12/) will be used for preprocessing of fMRI data. An alpha value of 0.05 will be used for all analyses, and Bonferroni-corrections will be applied when appropriate for controlling the type-1 error rate.

### Interim analyses {21b}

We do not plan to perform an interim analysis in the present study.

### Methods for additional analyses (e.g. subgroup analyses) {20b}

No additional analyses, including subgroup or adjusted analyses, are planned for this study.

### Methods in analysis to handle protocol non-adherence and any statistical methods to handle missing data {20c}

Protocol non-adherence will be addressed using an intention-to-treat analysis, which includes all participants as originally allocated regardless of adherence. Missing data will not be imputed. Missing data will be reported in the manuscripts. Sensitivity analyses will be conducted to evaluate the impact of different missing data assumptions, such as missing not at random.

### Plans to give access to the full protocol, participant-level data and statistical code {31c}

We plan to make the dataset and the statistical code available on the Open Science Foundation (OSF) after the study is published.

## Oversight and monitoring

### Composition of the coordinating centre and trial steering committee {5d}

This trial will be conducted in a manner consistent with good clinical practice. Principal Investigator Per M. Aslaksen is responsible for the overall conduct of the trial. Aslaksen coordinates the daily operations of the study in cooperation with the principal investigator and project manager at UIT, Marte C. Ørbo the responsible MD. Ole K. Grønli. Aslaksen and Ørbo oversee all research activities daily. Group meetings are held monthly. Only trained healthcare personnel interact with the participants.

### Composition of the data monitoring committee, its role and reporting structure {21a}

A Data Monitoring Committee has not been established. There is reasonable evidence for the effect and safety of the iTBS protocol used and the superiority of active compared to sham iTBS.

### Adverse event reporting and harms {22}

Any serious adverse events will be reported to the Regional Research Ethics Committee of Northern Norway and the University Hospital of North Norway immediately. If any participant suffers harm from participating in this study, they will receive medical treatment as required as a public patient.

### Frequency and plans for auditing trial conduct {23}

The study is conducted in accordance with good clinical practice and in accordance with the recommendations from the Regional Research Ethics Committee, Northern Norway. Participants in the study are cared for by experienced healthcare professionals bound by confidentiality. All data will be stored on secure network locations governed by the university hospital accessible only to project group members and protected by two-factor identification. The data management plan for this project was reviewed and approved by the Regional Research Ethics Committee, Northern Norway and the data protection officer at UNN-HF.

### Plans for communicating important protocol amendments to relevant parties (e.g. trial participants, ethical committees) {25}

In the event of protocol changes, the Regional Research Ethics Committee, Northern Norway will be notified in advance of making changes and updates will thereafter be published in ClinicalTrials.gov.

### Dissemination plans {31a}

The primary audience for this project includes scientific communities involved in psychiatry, clinical psychology, neuroscience, and non-invasive brain stimulation. Additionally, organizations in the field of psychiatry and stakeholders within the healthcare sector are considered valuable recipients of the project’s outcomes. Given the significant societal impact of innovative depression treatments, the general public is also regarded as an important audience.

The research findings will be disseminated through publication in peer-reviewed, open-access scientific journals. Collaborating closely with the media department at UiT, we aim to publicly share the results and provide general information about the project. Furthermore, the research group plans to actively participate in relevant scientific conferences and intends to apply for hosting symposiums focused on iTBS and depression treatment.

## Discussion

The present study protocol outlines the scientific rationale for a randomized, double-blind, parallel-group, sham-controlled neuroimaging trial. This trial aims to investigate the neurocognitive effects of intermittent theta burst stimulation (iTBS) on unipolar depression in patients referred to the psychiatric outpatient clinic at the University Hospital of North Norway.

Understanding the neurocognitive changes induced by iTBS is crucial for several reasons: first, to elucidate the mechanisms underlying iTBS; second, to determine how cerebral changes after iTBS relate to cognitive and antidepressant outcomes post-treatment and third; to inform patient selection for a therapy that holds promise for improvement of cognitive control functions in depression.

To date, few studies have assessed how iTBS affects cerebral grey or white matter structural integrity through pre- and post-treatment MRI examinations. This neuroimaging study boasts several strengths, including a placebo control and a relatively large sample size.

The study has the potential to provide novel scientific insights into the treatment effects of iTBS, a promising and currently considered safe treatment option for depression. Furthermore, it will be among the few studies to elucidate the neurocognitive mechanisms of iTBS and its potential to enhance neurocognitive function in depression.

## Trial status

This manuscript is based on the trial protocol dated August 13th 2024. At the time of submission, patient recruitment has begun, and it is estimated to be completed by 30 June 2025.

## Authors’ contributions {31b}

PMA is the principal investigator of this study at the UNN-HF. He developed the study design in collaboration with MCØ and TV. MCØ is the principal investigator at the UiT, the Arctic University of Norway. PMA and MCØ developed the funding application. The first draft of this manuscript was written by MCØ. TV is responsible for the brain imaging protocol. OKG is the responsible medical doctor. PMA, SH, and GC have made substantial contributions to this manuscript by revising it for intellectual content. All authors have edited the manuscript and approved the final draft.

## Funding {4}

This trial is funded by the Northern Norway Regional Health Authority, grant number HNF1671-23; email: forskningsmidler@unn.no. The funder has no role in the collection, management, analysis, and interpretation of the data; writing of the report; or decision to submit the report for publication and will not have authority over any of these activities. Open access funding is provided by UiT The Arctic University of Norway and the University Hospital of North Norway.

## Data availability {29}

All publications will be open access as required by the funding agency. The data management plan for this project was reviewed and approved by REC, UIT and UNN-HF. The complete data file with anonymized data will be stored at the UIT open data repository (https://dataverse.no) after the publications from this trial are published. Authorship will follow the Vancouver guidelines.

## Abbreviations

iTBS: Intermittent theta burst stimulation
HF rTMS: High-frequency repetitive transcranial magnet stimulation
LDLPFC: Left dorsolateral prefrontal cortex
ACC: Anterior cingulate cortex
RACC: Rostral anterior cingulate cortex
RMT: Resting motor threshold
MRI: Magnetic resonance imaging
fMRI: Functional magnetic resonance imaging
FA: Fractional anisotropy
RCT: Randomized controlled trial
WM: Working memory
EF: Executive functions
MADRS: Montgomery Aasberg Depression Rating Scale
BDI-II: Beck Depression Inventory II
DTI: Diffusion tensor imaging
LMM: Linear mixed model
ANOVA: Analysis of variance
ANCOVA: Analysis of covariance
GLM: General linear model
LMM: Linear mixed models
MNI: Montreal Neurological Institute
M.I.N.I.: Mini-International Neuropsychiatric Interview
UIT: University of Tromsø – The Arctic University of Norway
UNN-HF: University Hospital of North Norway – Health Trust
PALM: Permutation Analysis of Linear Models
RMT: Resting motor threshold
FS: FreeSurfer
ICC: Intraclass correlation
MCID: Minimal clinical important
BOLD: Blood oxygen level dependent
